# Atrial Fibrillation Ablation: A Single Center Comparison Between Remote Magnetic Navigation, Cryoballoon and Conventional Manual Pulmonary Vein Isolation

**Published:** 2010-12-26

**Authors:** Antonio Sorgente, Gian Battista Chierchia, Lucio Capulzini, Yoshinao Yazaki, Andreas Muller-Burri, Fatih Bayrak, Andrea Sarkozy, Carlo de Asmundis, Gaetano Paparella, Brugada Brugada

**Affiliations:** 1 Heart Rhythm Management Centre, UZ Brussel-VUB, Brussels, Belgium; 2 Department of Cardiology, University of L'Aquila, L'Aquila, Italy

**Keywords:** atrial fibrillation, ablation, remote magnetic navigation, cryoballoon

## Abstract

**Background:**

The aim of the study was to compare in our center the effect of different ablation techniques on intermediate term freedom from atrial fibrillation (AF) or atrial tachycardia (AT) in patients affected by refractory AF.

**Methods and Results:**

We retrospectively selected 94 patients who underwent AF ablation in our electrophysiological laboratory from June 2007 to December 2009. 29 patients underwent manual circumferential pulmonary vein isolation (mCPVI), 35 underwent remote magnetic navigation assisted CPVI (rmtCPVI) and 30 cryoballoon CPVI (cCPVI). Antiarrhythmic drugs were systematically stopped 2 months after the procedure (end of the "blanking period").

At a mean follow-up of 12,64 ± 6,41 months (range 2-31), the success rate for mCPVI group was 65.5% (19 patients), 66.7 % (20 patients) for the rmtCPVI group and 65.7 % (23 patients) for the cCPVI group (p = 0.625). Procedural and fluoroscopy times were significantly reduced in the cCPVI group (both p < 0.001). Univariate Cox regression showed that no clinical variables were independently associated with recurrence.

**Conclusions:**

In our center's experience cCPVI and rmtCPVI have been demonstrated to be as effective as mCPVI. cCPVI seemed to be associated with lower procedural and fluoroscopy times.

## Introduction

Since the demonstration that pulmonary vein (PV) ectopic activity is the main trigger of paroxysmal atrial fibrillation (AF) [1,2] percutaneous transcatheter ablation of this arrhythmia has become an increasingly used procedure for the treatment of this arrhythmia. Today, isolation of the PVs represents am established approach when performing AF ablation [1,2]. Traditionally, PV isolation (PVI) is achieved with a 'point by point' ablation technique by means of radiofrequency (RF) energy. Recently, novel technologies such as remote magnetic navigation (RMT) ablation and cryoballoon ablation have been demonstrated to be feasible and reliable in achieving PVI [3,4].

Three dimensional electroanatomically guided conventional manual RF ablation (mCPVI), 3D electroanatomically guided RMT RF ablation (rmtCPVI) and cryoballoon ablation (cCPVI). Compared to mCPVI, rmtCPVI and cCPVI might offer advantages in terms of procedure reproducibility and operator comfort. RmtCPVI enables the operator to direct in virtually any direction a flexible mapping catheter in the heart by the means of the movements of a computer mouse. This is rendered possible by slight variations in the magnetic field created by 2 activated magnets positioned at the patients side. The movements of the catheter are precise and the operator comfort optimal. The cCPVI technique permits PVI in more than 90% of veins [3]. The technique consists of inflating an over the catheter balloon in the LA and then wedging it in the PV ostium. Once total occlusion is demonstrated by the means of dye injection, cryoablation application can be started. The balloon will tightly adhere to the tissue during cryoablation. This might lead to better outcome when ablating particular anatomical structures, such as the ridge between the LSPV and the LAA by offering better contact. This technology permits a straightforward approach to atrial fibrillation ablation that might be less affected by operator skills as compared to the traditional point by point approach with a focal tip catheter.

Moreover, these new techniques might also offer better procedure reproducibility, reduce radiation exposure time and operator fatigue when compared to manual conventional PVI. However, a comparison of the results following PVI with these different techniques is lacking.

The aim of our study was to compare the short-term and mid-term outcome of PVI using three distinct technologies: RMT ablation, cryoballoon ablation and conventional manual ablation technique.

## Methods

We retrospectively analyzed all the patients referred to our department for documented drug refractory symptomatic AF that underwent a single PVI procedure between June 2007 and December 2009. To ensure a reasonable comparison of the 3 techniques and to diminish the influence of a learning curve on outcome, only operators' experience with > 30 patients using each technique was included and the first 5 patients undergoing PVI with each technique were excluded from our analysis. Procedural end point in all procedures was considered achievement of electrophysiologically proven PVI. Paroxysmal AF was defined as self-terminating AF episodes lasting less than 7 days. Persistent AF was defined as AF episodes lasting > 7 days or requiring pharmacological or electrical cardioversion because of intolerable symptoms. Permanent AF was defined when AF was the sole rhythm for the last 6 months. As mentioned above, 3 techniques were used to treat these patients: mCPVI, rmtCPVI and cCPVI. Patients were excluded from our retrospective analysis if they had already undergone a previous AF ablation procedure, in case of the occurrence of an acute complication that impeded procedure termination, in case of cross-over from rmtCPVI to mCPVI, in case additional "touch-up" applications with a focal catheter if PVI could not be reached using solely the cryoballoon, if additional linear ablation or ablation of complex fractionated atrial electrograms (CFAE) was performed, in case of absence of follow-up and if the patient already participated in the past and/or was included in another ongoing study in our center.

All ablations were performed under general anesthesia. Prior to the procedure, all patients underwent a 2D transthoracic echocardiogram (TTE) to evaluate left ventricular ejection fraction and to exclude any structural and/or valvular disease. A cardiac computed tomography (CT) and a transesophageal echocardiography (TEE) were performed the day before ablation in order to evaluate left atrial and PV anatomy and to exclude the presence of intracardiac thrombi.

### Ablation procedure

#### mCPVI procedure

A 6F pigtail catheter was positioned in the aortic root via a left femoral approach, to monitor arterial pressure and to assess the radiological position of the aorta. A 6F quadripolar or decapolar catheter were inserted in the right jugular vein or in the left femoral vein and advanced into the coronary sinus (CS). A double transseptal puncture was then performed under fluoroscopic guidance, using the right femoral venous approach. Following this, the 2 transseptal sheaths were advanced over the guide wires into the left atrium (LA). After gaining double left atrial access, a 70 UI/Kg heparin iv bolus was given. A selective angiogram in each PV in order to delineate the ostium was then performed. Next, a circumferential mapping catheter (Lasso™, Biosense Webster Inc., Diamond Bar, CA, USA) was introduced into the proximal PVs to gather baseline electrical information. Three-dimensional left atrial maps were reconstructed using a non-fluoroscopic navigation system (CARTO™, Biosense Webster Inc., Diamond Bar, CA, USA). Maps were acquired during sinus rhythm or AF. After map completion, RF energy was applied through a 3.5 mm irrigated tip ablation catheter (Navistar, Thermocool™, Biosense Webster, Inc., Diamond Bar, CA, USA) in a power-controlled mode with a power limit of 35 Watts and a maximum temperature of 48ºC. Each application lasted for a maximum of 60 s. Power was decreased to 25W when ablating on the posterior wall in order to avoid oesophageal injury. Ablation was continued at each point until the amplitude of the bipolar electrogram was reduced by 80% or decreased below 0.2 mV. No additional ablation lines were performed. The ablation strategy was to create contiguous focal lesions at a distance > 5 mm from the ostia of the PVs, thereby forming a circumferential line of conduction block around ipsilateral PVs with a circumferential mapping catheter (Lasso™, Biosense Webster Inc., Diamond Bar, CA, USA) placed in the PV ostium for PV potentials mapping. If PVI could not be achieved after completion of the circumferential ablation, further targeting the electrical breakthroughs on the ablation line was performed until complete isolation could be documented.

#### cCPVI procedure

A 6F pigtail catheter was positioned in the aortic root via a left femoral approach, to monitor arterial pressure and to assess the radiological position of the aorta. A 6F quadripolar or decapolar catheter was inserted in the right jugular vein or in the left femoral vein and advanced to the CS. A single transseptal puncture was then performed under fluoroscopic guidance, using the right femoral venous approach. After gaining left atrial access, a 70 UI/Kg heparin iv bolus was given. A selective angiogram in each PV in order to delineate the ostium was then performed. Immediately after, a 0.032" 260 cm long guide-wire (Emerald, Cordis, Johnson & Johnson, Diamond Bar, California, USA) was advanced in the left superior pulmonary vein (LSPV) and a steerable 15 F over-the-wire sheath (FlexCath, Cryocath, Montreal, Quebec, Canada) was positioned in the LA. A circular mapping catheter (Lasso, Biosense Webster, Inc., Diamond Bar CA, USA) was then advanced in each PV ostium to obtain baseline electrical information. After withdrawing the mapping catheter, a 28 mm double walled cryoballoon (Arctic Front, Cryocath, Montreal, Quebec, Canada) was advanced over the wire up to the LA, inflated and positioned in the PV ostium of each vein. Optimal vessel occlusion was considered to have been achieved when selective contrast injection showed total contrast retention with no flow to the atrium. For each vein, cryoablation consisted of a minimum of 2 applications lasting 5 minutes each. Whenever possible, we tried to engage with the guide-wire 2 different branches of the same vein and to orient the balloon differently, in the attempt of covering a wider ostial surface. However, if successful occlusion could only be obtained in one branch, both applications where delivered by leaving the guide-wire in the same branch. Usually, the LSPV was treated first, followed by the left inferior (LIPV), right superior (RSPV) and right inferior (RIPV). In order to avoid phrenic nerve palsy, a complication observed during RSPV isolation with CBA, a quadripolar catheter was inserted in the superior vena cava and diaphragmatic stimulation was achieved by pacing the ipsilateral phrenic nerve with a 1000 ms cycle and a 12 mA pulse output. The reason of pacing at such a slow rate was to prevent catheter displacement in the early phases of application due to diaphragmatic contraction. After ablation in all PVs, the circular mapping catheter (Lasso, Biosense Webster, Inc., Diamond Bar CA, USA) was then placed in each ostium to evaluate isolation. In case of persistence of PV potentials extra cryoballoon applications were performed until complete PVI.

#### rmtCPVI procedure

The procedure was performed as in the manual RF ablation group with the exception that left atrial electroanatomic mapping and RF ablation were performed by using an integrated CARTO RMT system (Biosense-Webster, Inc., Diamond Bar CA, USA) together with the Niobe II remote magnetic system. CARTO-RMT integration procedure, magnetic navigation procedure and remote mapping were identical to those previously described by Pappone and coworkers [4], except that we used an irrigated 3.5 mm ablation catheter (Thermocool RMT NaviStar, Biosense Webster, Diamond Bar, California) instead of a 4 mm flexible catheter (NaviStar-RMT, Biosense Webster, Diamond Bar, California). The ablation protocol was identical to conventional manual RF ablation (see description above).

### Follow-up and postablation clinical strategy

2D TTE was repeated the day after the procedure in order to evaluate the mitral valve and the left ventricular ejection fraction, and to exclude the presence of pericardial effusion. All patients were discharged on oral anticoagulation with a target international normalized ratio between 2 and 3 and on antiarrhythmic drugs previously ineffective. Warfarin was continued for a minimum of 3 months after the ablation procedure and maintained afterwards according to the prevalent rhythm and CHADS2 score of the patients. Antiarrhythmic drugs were stopped after a blanking period of 2 months. Ambulatory electrocardiographic recorders were used to correlate symptoms with arrhythmia recurrence. All patients enrolled underwent physical examination and Holter recordings at 1, 3, 6 months and 1 year after the procedure and in case of the occurrence of arrhythmic symptoms.

### Primary endpoint

Ablation was considered successful in the absence of symptomatic or asymptomatic atrial tachyarrhythmias lasting ≥ 30 s, identified on surface ECG, Holter monitoring or 7-day ECG recording, without antiarrhythmic drug therapy.

### Statistical analysis

Continuous variables are expressed as mean values ± standard deviation, and were, when appropriate, compared using the Student's t-test. Categorical variables are expressed as numbers and percentages and were, when appropriate, compared with the Pearson x^2^ analysis or Fisher's exact test. Predictive analysis of recurrence over the follow-up time was assessed through the Cox regression method. The multivariate model was used adjusting for AF type, sex, age ≥ 65 years, left atrial dimensions, AF duration before the index procedure, antiarrhythmic drugs, CAD, and procedure assignment. The hazard ratio (HR) and its 95% confidence interval (CI) were retrieved from the model. Kaplan-Meier event-free survival analysis was conducted to assess the cumulative freedom from recurrence. A p value < 0.05 was considered statistically significant. SPSS, version 11.0 (SPSS, Inc., Chicago, IL, USA) was used for statistical analysis.

## Results

A total of 267 patients affected by symptomatic drug refractory AF consecutively referred for an ablation to our department were screened for eligibility at our retrospective analysis. Of these patients, 80 were excluded because they had already undergone a previous AF ablation procedure in another center, 3 because of the occurrence of an acute complication that impeded procedure termination, 25 because of cross-over from rmtCPVI to mCPVI, 12 because of cross-over from cCPVI to mCPVI, 15 because during ablation additional linear ablation or ablation of complex fractionated atrial electrograms (CFAE) were performed, 30 because of impossibility to retrieve information about their follow-up and 8 because they were already included in another ongoing study in our center.

**Figure 1:**
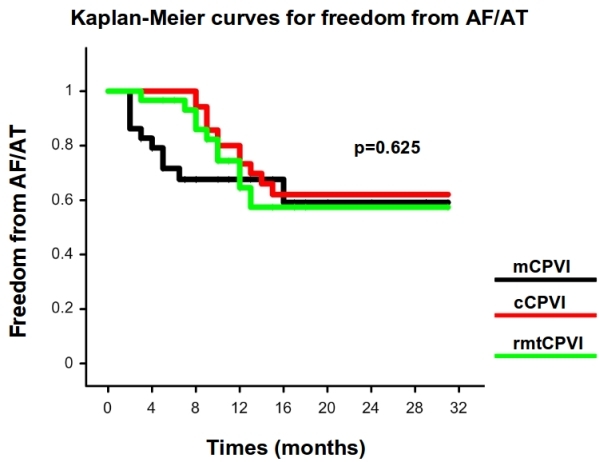
Kaplan-Meier estimation of the time to atrial tachyarrhythmia recurrence after ablation in the 3 groups (mCPVI: black line, cCPVI: red line, rmtCPVI: green line).

**Figure 2:**
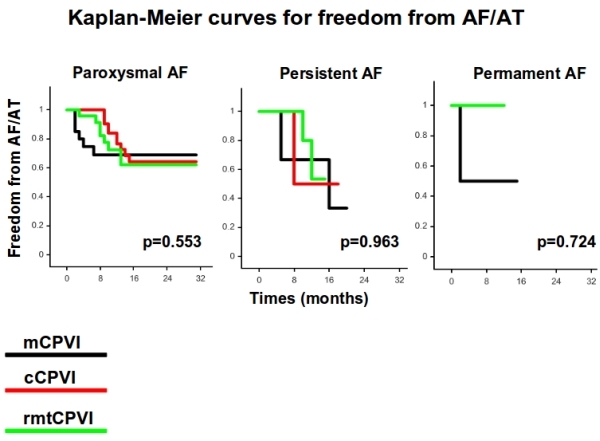
Kaplan-Meier estimation of the time to atrial tachyarrhythmia recurrence after ablation in the 3 groups according to the AF type (mCPVI: black line, cCPVI: red line, rmtCPVI: green line).

**Figure 3:**
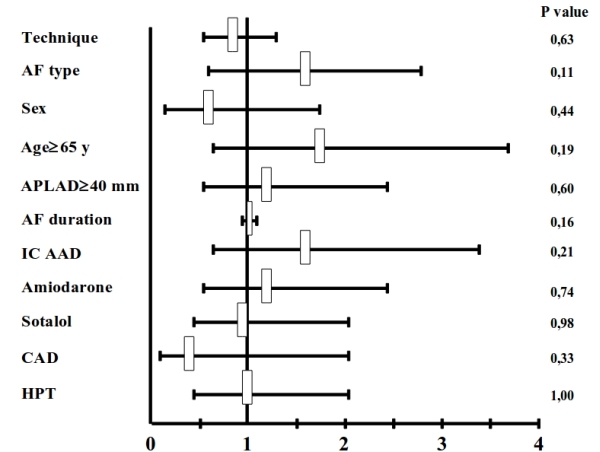
Hazard ratio from univariate Cox model of event-free survival. AF: atrial fibrillation; APLAD: anteroposterior left atrial diameter; IC: IC class antiarhhythmic drugs; CAD: coronary artery disease; HPT: arterial hypertension.

94 patients (79 males, 55.9 ± 9.8 years) were finally considered in our retrospective analysis. All patients had symptomatic and drug-resistant AF (75 paroxysmal, 16 persistent and 3 permanent). 92 patients underwent 24 hour Holter recordings at the time frame of 1, 3, 6 and 12 months. Only 2 (1 in the group of the mCPVI and 1 in the group of cCPVI) underwent a 7 days ECG recording at the correspondent time period.  had Of them, 29 patients underwent mCPVI, 30 cCPVI and 35 rmtCPVI. The baseline characteristics of the patients are shown in Table 1. No significant differences among the 3 groups in terms of sex, age, AF duration, LA size and left ventricular EF were present. Follow-up was slightly longer in patients that underwent cCPVI compared to patients who underwent mCPVI and rmtCPVI (p = 0.039). Procedural time and fluoroscopy time were significantly shorter in the cCPVI group (both p < 0.001). The procedural endpoint was achieved in every patient enrolled in the study (100%). At a mean follow-up of 12.64 ± 6.41 months (range 2-31), the success rate without antiarrhythmic therapy for mCPVI group was 65.5% (19 patients), 65.7 % (23 patients) for the cCPVI group and 66.7 % (20 patients) for the rmtCPVI group (p = 0.625). The event-free survival estimates for the 3 groups are reported in Figure 1. The event-free survival estimates for the 3 groups according to the AF type are displayed in Figure 2. Univariate Cox regression showed that no clinical variables were independently associated with recurrence (Figure 3).

### Complications

A left femoral artery pseudoaneurysm was found in 4 patients the day after the index procedure (2 in the mCPVI group, 1 in the cCPVI group and 1 in the rmtCPVI group). An arteriovenous fistula which required vascular intervention and a 2-day prolongation of hospitalization stay occurred in one patient of the mCPVI group. In the same group, moderate asymptomatic pericardial effusion was documented in 1 patient the day after procedure. Serial echocardiographic examinations showed complete resolution after 1 month. Transient phrenic nerve palsy was observed in 3 patients when delivering cryothermal energy in the RSPV. Diaphragmatic contraction completely recovered before the end of the procedure. No cerebrovascular accident occurred during or after any procedure.

## Discussion

To our knowledge this is the first single center study that compares the outcome of AF ablation following circumferential PVI with 3 different techniques.

The main findings of our study are the following:

1. At a mean follow-up of 1 year, approximately 65% of patients were free of symptomatic atrial arrhythmias;
2. There was no significant difference in terms of success rate among the 3 compared groups;
3. cCPVI proved to be associated with significantly shorter procedural times and lower X ray exposure;
4. No baseline population characteristics appeared to predict AF recurrence.

The overall proportion of patients free of symptomatic atrial arrhythmias at an average 1 year follow-up after a single PVI procedure was approximately 65%. This percentage is in line with previously published studies that report success rates of one single procedure of AF ablation ranging between 56 and 88% [5-10]. This reasonably high percentage of arrhythmia free patients following PVI procedure highlights once more the pivotal role played by these anatomical structures in the genesis and maintenance of AF [1]. Furthermore, our population also included persistent and permanent variants of the arrhythmia which are known to be associated with lower success rates after ablation [10,11]. Success rate might have been higher if only patients affected by paroxysmal AF had been included.

Interestingly, there was no significant difference in success rate in the 3 groups. Our observation confirmed the efficacy of all 3 technologies in achieving PVI as already reported in the literature [1-4]. Recent studies aiming at comparing different technologies for AF have been published. Linhart et al [12]i reported similar findings when comparing cryoballoon with point by point RF PVI. In a relatively small study 40 patients were randomised to either cryoballoon ablation or manual RF ablation. At a follow-up of approximately 12 months roughly 50% of patients were free of AF and there was no significant difference in the 2 groups in terms of outcome.

Recently Di Biase et al [13] described their experience in a large cohort of patients with the Hansen robotic navigation system in comparison to manual AF ablation. The authors concluded that robotic navigation system is as safe and effective as its manual counterpart. However, physical strain and X-ray exposure was dramatically reduced resulting in enhanced operator comfort, when the ablation was performed with the Hansen system. Up to date no prospective data comparing conventional manual with rmtCPVI ablation using an irrigated 3.5 mm tip RF catheter have been published. In our study, cCPVI had significantly shorter ablation procedure duration and lower X-ray exposure times as compared to the other 2 approaches. This might be explained by various factors. First, in the cCPVI group with the exception of 1 patient who exhibited a left sided common ostium, all individuals had 4 distinct PV ostia. No patient had accessory veins. "Straightforward" anatomies might prove easier when performing PVI with "single" shot devices with consequent lower procedural times. As mentioned before, by its own nature cryoenergy tightly "sticks" to the tissue in contact. Thus, after demonstration of successful occlusion, cryoapplication can be started without the need of X-ray monitoring during most of the lesion formation time, as the cryoballoon will not loose its original position. This might give an explanation to the lower fluoroscopic times observed in the cCPVI group. Secondly, compared to the already published reports [3,4] in our center procedural and fluoroscopy times observed in the rmtCPVI and mCPVI groups were significantly higher. This finding can be explained by the fact that all the procedures were performed with the help of electrophysiological fellows and this can have increased procedural times and fluoroscopy times in the rmtCPVI and mCPVI groups.

Finally, in our study no baseline clinical parameters were predictors of failure to maintain sinus rhythm. This is line with a recent observation by Bertaglia and co-workers [15]. In our study, the rate of success at 12 months follow-up was similar among patients with paroxysmal, persistent and permanent AF. This result can be explained considering first of all a possible misclassification of paroxysmal atrial fibrillation as persistent atrial fibrillation occurred in a few selected cases (for example patients with episodes of AF shorter than 7 days, who underwent electrical or pharmacological cardioversion due to symptoms or hemodynamic instability) and secondly the relatively small mean dimensions of the LA in our study population (41 mm).

## Study limitations

The most important limitation of our study is its retrospective and observational design. A randomized prospective study is actually ongoing in our center to add more data and to confirm the above described findings. Nevertheless, although only a small portion of the totality of PVI performed in our center was included in our analysis, it corresponds to a natural population of AF patients referred to our electrophysiological laboratory and gives a realistic picture of the mid-term clinical results of 3 different techniques for atrial fibrillation ablation. Secondly the inference to general population is strongly limited by the single-center design of the study. Thus results might not be generalized.

## Conclusions

RMT guided RF, manual conventional RF and cryoballoon ablation are all effective in producing PVI. Furthermore all 3 techniques have similar rates in terms of freedom of AF after 1 year follow-up with success rates approximating 65%. However, cryoballoon ablation might be faster and might expose the operator and the patient to lower irradiation as compared to the other 2 approaches. No significant predictor of AF recurrence following ablation was observed in our population.

## Figures and Tables

**Table 1:**
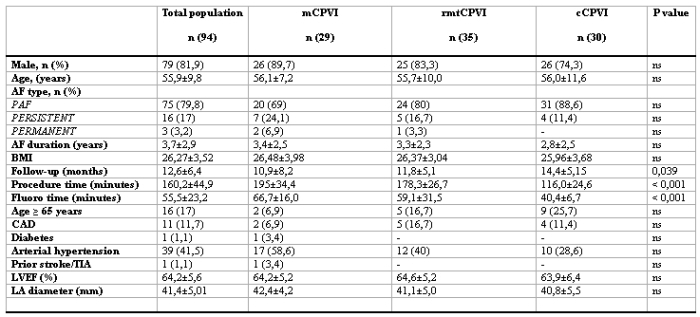
Baseline characteristics  of the patients

PAF: paroxysmal atrial fibrillation, PERSISTENT: persistent atrial fibrillation, PERMANENT: permanent atrial fibrillation, CAD: coronary artery disease, TIA: transient ischemic attack, P < 0.05 was considered as statistically significant.
